# Factors influencing within-group conflict over defence against conspecific outsiders seeking breeding positions

**DOI:** 10.1098/rspb.2018.1669

**Published:** 2018-12-19

**Authors:** Susanne Schindler, Andrew N. Radford

**Affiliations:** School of Biological Sciences, University of Bristol, Bristol, UK

**Keywords:** animal groups, breeding position, defence, inclusive fitness, intrusions, subordinate contributions

## Abstract

In social species, groups face a variety of threats from conspecific outsiders. Defensive actions are therefore common, but there is considerable variation in which individuals contribute and to what extent. There has been some theoretical exploration of this variation when the defence is of shared resources, but the relative contributions when a single intruder threatens a particular breeding position have received less attention. Defensive actions are costly, both for the individual and dependent young, and contributions are likely to differ depending on individual sex, rank and size, current breeding stage, infanticide risk and relatedness levels. Here, we model analytically the relative fitness benefits of different group members to engaging in defence against individual intruders and determine when within-group conflicts of interest might arise over these defensive contributions. Conflicts of interest between the challenged breeder and other group members depend on relatedness to the brood and the potential relatedness reduction if an intruder acquires breeding status. Conflicts are more likely to occur when there is a low chance of winning the contest, low infanticide rates, inefficient defence from helpers, a long remaining brood-dependency period and high external (non-contest-related) mortality. Our work can help explain variation in defensive actions against out-group threats.

## 1. Introduction

In social species from hymenopterans to humans, relatively stable and permanent groups form for a variety of reasons [1]. These groups and their members often face threats from rival conspecific groups and individuals [2–5]. When the out-group threat is to shared resources such as food or the whole territory [2,3,6], there may be clear costs of losing for all or most group members and so an incentive for joint defence. Despite this, collective-action problems [7] can lead to some variation in the amount each group member commits to resource defence [8,9]. When the out-group threat is to a breeding position, whereby an individual intruder is seeking to usurp a particular existing breeder [4,5,10], the interests of group members are even less likely to be perfectly aligned, and considerable within-group variation exists in defensive contributions [10,11]. Explaining the factors influencing this variation in group defensive actions is fundamental to a full understanding of social evolution.

Precisely which group members contribute to defence against an individual intruder, and how much they contribute, will differ depending on a range of costs and benefits relating to individual sex, rank and size, current breeding stage, infanticide risk and relatedness levels. Defensive actions are costly: when contests escalate to violence, injury or even death can ensue [12]; all exchanges with outsiders, whether signalling or physical, take time and energy, reducing current and future foraging and parental-care opportunities [13,14]. There are also clear potential costs from losing a contest with an individual intruder, ranging from loss of the breeding position for the same-sex breeder [11,15] to loss of the current brood owing to infanticide [16]; there are concomitant benefits from winning such contests, and thus preventing these actions. Each individual must therefore trade-off the relevant costs and benefits when deciding how much to contribute to defence on a given occasion, and that trade-off will differ between group members. For example, while the replaced breeder loses all future mating chances, the other breeder can continue to reproduce with the intruder; aggression towards intruders is therefore typically sex-specific [11,17], but can also be influenced by social rank [3,18]. The size and condition of individuals should also affect decisions about defence participation, with those who are larger and in a better condition likely to contribute more [3,14]. Similarly, the age of any current offspring will probably influence the trade-off, as older offspring may be less vulnerable to temporary reductions in parental care or to infanticidal attacks [16]. Finally, relatedness will be important, with those group members losing most from future reproduction by an unrelated intruder expected to contribute more to defence against them [3,19]. While there have been empirical studies considering each of these factors, understanding their interplay is more complex.

Theoretical modelling can help to generate testable predictions about the variation in within-group contributions to defensive actions when a breeding position is threatened by an outsider. To date, models of social behaviour have focused on either the causes of group formation or helping [20–22], or have predicted individual levels of parental care [23–26], reproductive skew [27,28] or defence of a common good [29]. For the case of defending a common good, Gavrilets & Fortunato [30] have used the adaptive-dynamics framework and individual-based simulations to consider contributions to joint defence with respect to the collective-action problem. Their model led to two major predictions. First, dominant group members that can secure a higher share of resources within a group should engage substantially more in collective defence than lower-ranked group members. Second, individuals should increase their defence contribution with higher within-group relatedness. However, the trade-offs in costs and benefits will probably differ when the threat is to an individual breeder. Here, we focus on the situation when a breeder's position is threatened by an individual outsider and quantify when social defensive behaviour ceases to be beneficial for group members.

Inclusive fitness is a powerful concept to understand the evolution of social behaviours and has been usefully applied in explaining the evolution of altruism [31,32], cooperation [33] and eusociality [34] (see [35] for an alternative viewpoint). The inclusive fitness of an individual summarizes its expected genetic contribution to the next generation based on its behaviour; an optimal action in an evolutionary sense is having the highest inclusive fitness value. Inclusive fitness can be calculated for several different actions; for example, whether an individual contributes to defence against an intruder and how much it contributes. Here, we use the inclusive fitness concept to calculate when conflict arises between group members over defensive responses to individual intruders seeking breeding status. We calculate inclusive fitness values for different group members—two breeders, one helper—and identify when interests diverge and, thus, when within-group conflict is likely to occur with respect to out-group contests.

## 2. Model

We propose an analytic model investigating the within-group consequences of interactions between a focal group and one intruding outsider trying to obtain a breeding position within the group. The model focuses on the consequences for two reproductive events: the current brood, produced by the breeders in the focal group prior to the interaction with the outsider; and the brood produced after that interaction with the outsider, when either the same breeders reproduce or the brood is produced by the remaining breeder and the outsider who has successfully obtained the other breeding position. We consider the effect of various factors—size differences between the breeders and the helper and between the breeders and the outsider, infanticide risk, within-group relatedness, duration of the remaining brood-dependency period and external mortality—on inclusive fitness for group members and thus the potential conflict between them.

The general situation that we model is commonly found in a wide range of species, including, but not limited to, cooperatively breeding vertebrates [36]. As just a few examples, species where groups can face an outside threat from single intruders include: birds such as Arabian babblers (*Turdoides squamiceps*) [37]; subdesert mesites (*Monia benschi*) [38], green woodhoopoes (*Phoeniculus purpureus*) [39] and pied babblers (*Turdoides bicolor*) [40]; fishes such as *Lamprologus brichardi* [41] and *Neolamprologus pulcher* [11]; and mammals such as African lions (*Panthera leo*) [42], Thomas langurs (*Presbytis thomasi*) [43] and black howler monkeys (*Alouatta igra*) [44]. In the small cichlid fish *N. pulcher*, for instance, territorial social groups consist of a dominant breeding pair, 1–20 sexually mature but non-breeding subordinate helpers of various sizes, and dependent offspring of the breeders [11,45,46]. Intrusions by out-group individuals can represent a threat to the position of existing similarly sized group members, including the breeders [46], but all group members can contribute to defence against conspecifics [11,45]. In African lions, prides consist of 1–9 adult males, 1–18 adult breeding and non-breeding females, and their dependent offspring [42]. Outsider males (including single individuals) compete to gain residence in prides and thus a reproductive position [42]; incoming males present an infanticidal risk, because they may attempt to kill cubs of resident females to bring the latter into oestrus sooner [47]. Consequently, both resident males and females may engage in defence against conspecific intruders [42,48].

### (a) Social group and intruder

**Figure 1.**
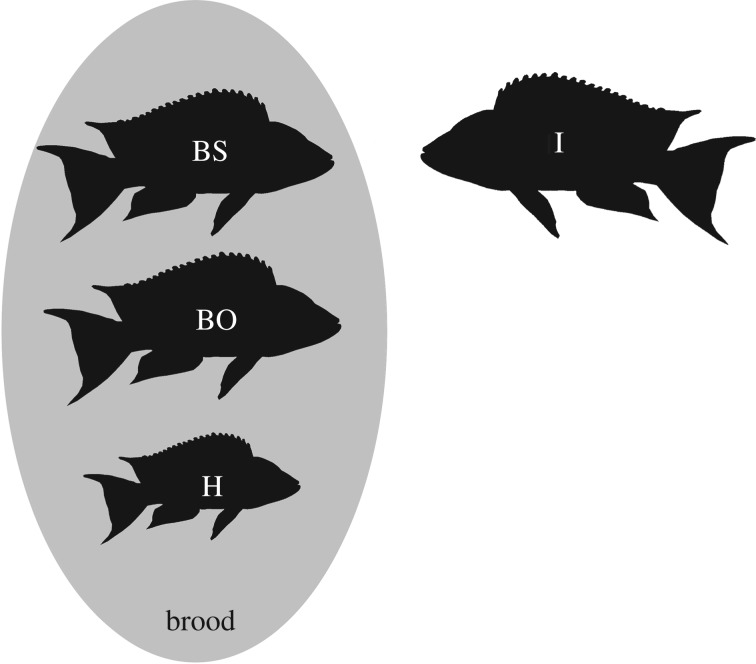
Group set-up in the example of a fish species. BS, the same-sex breeder as the intruder; BO, the opposite-sex breeder to the intruder; *H*, the helper and *I*, the intruder. BS and BO are equally sized. *H*'s size can range from half the size to the same size as the breeders. *I*'s size can range from the same size to 25% larger than the breeders. Silhouettes are based on photographs of *Neolamprologus pulcher* taken by Ines Braga Goncalves.

The focal group in our model consists of two breeders and one non-breeding helper (the simplest scenario), caring for a brood produced by the current breeders. The breeding pair comprises an individual of the same sex as the intruder (BS), and thus under direct threat of losing its breeding position, and an individual of the opposite sex to the intruder (BO); the intruder is denoted with *I* and the helper with *H* (figure 1).

We consider four different family relations between breeders and the helper:

(i) *H*_unrel_ is a helper who is unrelated to both BS and BO. The relatedness coefficient between the helper and the brood (*r*_unrel_) is 0;
(ii) *H*_O_ is either an offspring or sibling of BO, but not BS; in both cases, the helper's relatedness coefficient to the current brood (*r*_O_) is 0.25;
(iii) *H*_S_ is either an offspring or sibling of BS, but not BO; in both cases, its relatedness coefficient to the current brood (*r*_S_) is 0.25; and
(iv) *H*_SO_ is an offspring of the breeders; its relatedness coefficient to the current brood (*r*_SO_) is 0.5.

In cooperatively breeding species, for example, all four of these scenarios are common; there is variation within and between species in their frequency of occurrence [36,49]. Such cooperatively breeding groups are commonly founded on nuclear family units with the previous offspring of the breeders retained to help (*H*_SO_ scenario). Relatedness to the current offspring being helped can be reduced when one of the helper's parents dies, disperses or is replaced; a relatedness of 0.25 also arises when same-sex siblings disperse together but only one breeds (*H*_O_ and *H*_S_ scenarios). Helping by unrelated individuals is also common (*H*_unrel_ scenario); more so than previously recognized [49]. As a specific example, helper-to-brood relatedness coefficients have been shown to range from 0 to 0.5 in the cooperatively breeding cichlid *N. pulcher* [50].

The relative size difference between antagonists often influences contest outcome in nature [51], and intruders can be larger or smaller than breeders. However, intruders that are smaller than a breeder do not pose a threat, either because smaller intruders do not attempt a contest or, if they do, the contest is quickly settled in favour for the breeder with a low-cost signalling exchange. We therefore focus our model on intruders that are at least the size of the two breeders, which we assume are equally large. To capture a size advantage of the intruder over BS (and BO), we introduce the parameter *s_I_* (see the electronic supplementary material, table S1 for a full list of notations). This parameter can take non-negative values; for example, *s_I_* = 0 means that the intruder is the same size as BS and *s_I_* = 0.25 means that the intruder is 1.25 times the size of BS. Helpers are often smaller than the breeders, and so we allow the size of the helper to be smaller or up to the same size as the breeders (see parameter *k*_2_ below).

### (b) The contest

The intruder challenges the breeding position of BS and, if successful, replaces BS. This replacement occurs when the group's defence (the total amount of involvement in the contest provided by all group members) is below a threshold. BS, BO and *H* can all potentially participate in the contest; the current brood cannot participate. Defeating a very large intruder requires more involvement, so we assume that the threshold for winning, *φ*_min_(*s_I_*), increases with the intruder's size advantage *s_I_*:

2.1 $$\varphi_{\min}(s_{I})=\text{exp}[\text{ln}(\varepsilon )\text{exp}(-k_{1}s_{I})],$$

where *ɛ* is the minimal amount of involvement needed to defeat an intruder of the same size as the breeders. To capture the many scenarios of how fast the threshold of total group involvement increases with *s_I_*, we introduce parameter *k*_1_. This parameter can take any value above 1 (although values larger than 20 are probably unrealistic); the larger *k*_1_, the faster the threshold rises with size advantage *s_I_* (see the electronic supplementary material, A.1 and figure S1).

If the group wins, BO and BS produce a new brood; if the group loses, *I* replaces BS, and BO produces a new brood with *I*. When an intruder takes over a breeding position in a group, there is the possibility of infanticide, which we capture with the parameter *μ*. This parameter ranges from 0 (no infanticide) to 1 (all offspring of the current brood are killed). An intermediate value of *μ* either means that a fraction *μ* of the current brood is killed by the successful intruder or that the intruder kills all offspring with probability *μ*; both scenarios are mathematically equivalent. In the natural world, there is wide variation in infanticide rates [52]; for example, no sexually selected infanticide in grizzly bears (*Ursus arctos*) [53] or bats [54], some reported instances of infanticide in barn swallows (*Hirundo rustica*) [55], and experimentally estimated high rates (13–50%) of infanticide in collared lemmings (*Dicrostonyx groenlandicus*) [56]. In our model, infanticide does not reduce the time until the next reproductive bout because we are interested in the conflict between group members rather than in the best reproductive strategy of the intruder.

In the event of a takeover by *I*, the relatedness coefficients between the helper and future brood can change. Specifically, while *r*_unrel_ and *r*_O_ will stay the same, *r*_SO_ decreases from 0.5 to 0.25, and *r*_S_ decreases from 0.25 to 0.

### (c) Involvement in the contest

Each group member (apart from the brood) can defend the breeding position of BS against the intruder by choosing an amount of involvement in the contest, ranging from 0 (no involvement) to 1 (maximal involvement). Contest involvement carries a survival cost and we assume that survival decreases linearly with involvement. That means, an involvement of *φ* reduces survival by a factor of 1 − *φ*. If a group member gets involved in the contest at the maximal level (*φ* = 1), it sacrifices its life in the contest. Individual involvement is denoted with *φ*_BS_ for BS, *φ*_BO_ for BO and *φ_H_* for the helper.

Group members may vary in their effectiveness in defence depending on, for instance, their size and strength. To capture this potential difference in defence effectiveness between breeders and helpers, we introduce parameter *k*_2_. This parameter can take continuous values including and above 1; *k*_2_ = 1 means that breeders and helper are of the same size and equally effective in defence, while, for example, *k*_2_ = 2 means that helpers are half the size of a breeder and a helper's involvement is discounted by 0.5. In other words, if *k*_2_ = 2 then two units of a helper's involvement count as much as one unit of a breeder's involvement. As an example, in the cooperatively breeding cichlids *L. brichadi* and *N. pulcher*, helpers are about 2.5–5 cm long and breeders 6–6.5 cm long [41,57], which translates into *k*_2_-values between 1.2 and 1.6. In general, and for any setting of *k*_2_, the total group involvement amounts to $\Phi =\varphi_{\mathrm{BS}}+\varphi_{\mathrm{BO}}+(1/k_{2})\varphi_{H}$ (see the electronic supplementary material, A.2).

### (d) Intruder investment in the contest

The intruder must invest in the contest if it is to replace BS, and its investment should depend on its size advantage over BS. For instance, if *I* is very much larger than BS, its investment can be lower than when *I* and BS are of the same size. We distinguish between ‘investment’ (by the intruder) and ‘involvement’ (by group members) to avoid the misconception that comparing group involvement to intruder investment decides the contest outcome. Rather, the contest outcome is dependent on whether group involvement exceeds the threshold of minimal involvement needed and this threshold increases with the size advantage of the intruder (see above). We capture the impact of the size advantage of *I* over BS on the intruder's investment with the function *φ_I_*(*s_I_*):

2.2 $$\varphi_{I}(s_{I})=\frac{1}{1+(s_{I}/\varepsilon )}.$$

We thus assume that the intruder's investment decreases exponentially from 1 (when *s_I_* = 0, i.e. BS and *I* are of the same size) and converges to zero for very large values of *s_I_*; see the electronic supplementary material, A.3 and figure S2.

### (e) Dependence of brood

During and after a contest, the current brood suffers from lower survival for two potential reasons. First, because parental care is lower when group members are involved in the contest or need to recover from it; this cost is apparent regardless of the contest outcome. Second, because of the risk of infanticide if the intruder replaces BS. Both survival costs, however, are lessened with brood age; as the brood approaches independence, the remaining period of parental care is shorter and the likelihood of infanticide is lower. To capture the impact of remaining brood dependence on brood survival, we introduce the parameter *k*_3_ and assume a cost function, *F*(*c*, *k*_3_):

2.3 $$F(c,\;k_{3})=c+(1-c){\text{e}}^{-k_{3}},$$

where *c* captures the survival probability of dependent brood both during and after a contest (see the electronic supplementary material, A.4 for more details). The parameter *k*_3_ can take any value above and including zero and determines the rate at which brood costs decrease with decreasing time to independence (electronic supplementary material, figure S3). In biological terms, a value of *k*_3_ = 0 means that all the brood is independent; intermediate values of *k*_3_ (0 < *k*_3_ < 5) mean that some period of brood dependency is left; and for *k*_3_ ≥ 5, nearly the full duration of brood dependence remains.

### (f) External mortality

We assume that the breeders, the helper and the intruder have the same baseline survival rate. It is possible that smaller helpers are more vulnerable than larger breeders. However, if a helper faces lower survival chances then it might be even more reluctant to become involved in a contest, which increases the potential for conflicting interests within the group. Our assumption of equal survival rates is therefore conservative. In our model, we test several values of external survival rates, *p*_surv_, ranging from very low (*p*_surv_ = 0.1) to certain (*p*_surv_ = 1), the latter being equivalent to no external mortality. Among bird species, for example, survival rates vary hugely depending on a wide range of factors [58].

### (g) Inclusive fitness

The inclusive fitness values for both contest outcomes (lost or won), for all group members (BS, BO and *H*), and for all family relations (*H*_unrel_, *H*_O_, *H*_S_ and *H*_SO_) are derived and given in the electronic supplementary material, B. A conflict of interest within the group occurs when one group member would have higher inclusive fitness when the group defeats the intruder, while a different group member prefers a lost contest. The latter can arise if winning the contest requires such a large involvement that the individual's inclusive fitness value is higher when the contest is lost with zero involvement. We term the point in parameter space at which the inclusive fitness from losing the contest exceeds the value from winning as a ‘switch point’. For each group member, losing the contest can at times be better than winning (see the electronic supplementary material, C). However, the conditions for this to happen vary among the group members, such that there is a within-group conflict about contest involvement with the intruder.

## 3. Results

Each group member (except *H*_unrel_) can have a switch point, when it pays to change from participating in defence against an individual intruder seeking a breeding position to not participating in that defence. The position of the switch point varies among group members; however, the switch points for BS and *H*_S_, and those for BO and *H*_O_, coincide (electronic supplementary material, C.3).

**Figure 2.**
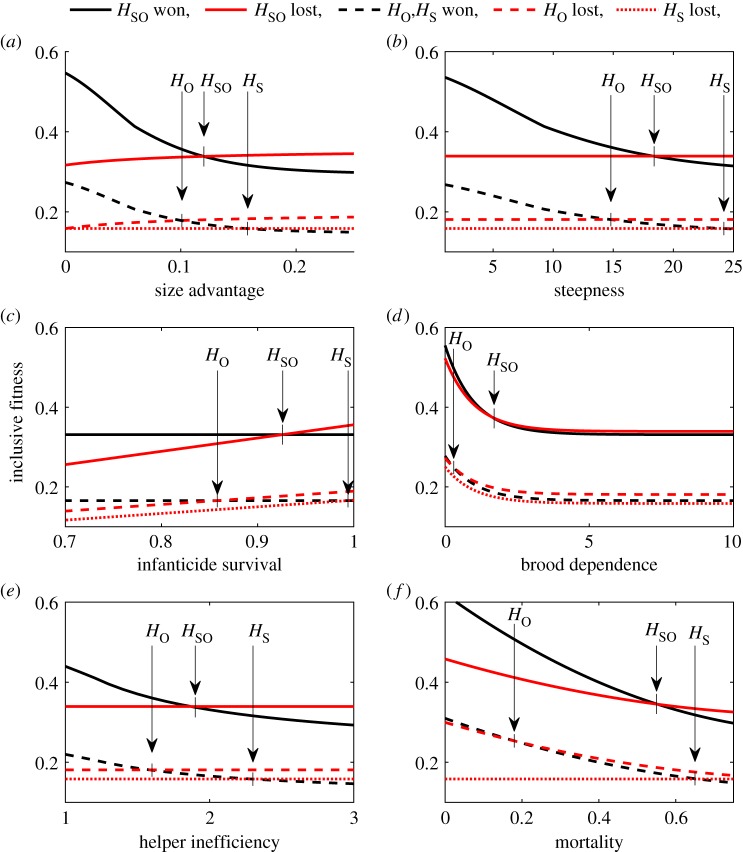
The helper's inclusive fitness when defending (becoming involved in the contest with the intruder) and defeating the intruder (black) and when not defending and the intruder takes over BS's position (red). Panels show inclusive fitness as functions of the (*a*) size difference between intruder and breeders (*s_I_*); (*b*) steepness of the threshold curve for involvement that is required to successfully defend against the intruder (*k*_1_); (*c*) probability of infanticide (*μ*); (*d*) duration of remaining brood dependence (*k*_3_); (*e*) helper inefficiency (*k*_2_); and (*f*) external mortality (1 − *p*_surv_). Solid lines refer to *H*_SO_, dashed lines to *H*_O_, and dotted lines to *H*_S_. In the case of defending and defeating the intruder, *H*_O_ and *H*_S_ can expect equal fitness returns (black dashes). Fixed parameters are: *s_I_* = 0.13, *μ* = 0.05, *k*_1_ = 20, *k*_2_ = 2, *k*_3_ = 10, *p*_surv_ = 0.4. (Online version in colour.)

**Figure 3.**
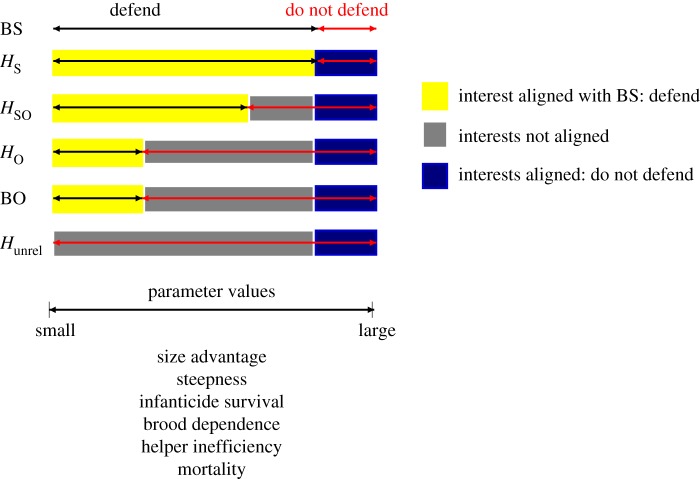
Schematic summary of positioning of switch points for each group member, depending on variation in the different parameter values.

Each panel in figure 2 shows the inclusive fitness returns of different group members as a function of one parameter. Moving from left to right, the sequence of switch points among group members is the same in all six panels: first *H*_O_ (and also BO), second *H*_SO_ and third *H*_S_ (and also BS). Thus, the switch points predict the loyalty of group members to BS (measured in size of parameter space where interests are aligned; figure 3): *H*_S_ is always loyal to BS, *H*_SO_ is somewhat less loyal, BO and *H*_O_ are even less loyal and *H*_unrel_ is least loyal. The ordering of switch points is driven by the relatedness of group members to the current brood and the drop in relatedness after a takeover: *r*_S_ decreases strongly (from 0.25 to 0, 100% reduction); *r*_SO_ decreases by the same extent but stays at a moderate level (from 0.5 to 0.25, 50% reduction); *r*_BO_ and *r*_O_ are unaffected by a takeover (they remain at 0.5 and 0.25 respectively, 0% reduction); and *r*_unrel_ is zero in any case. Loyalty is thus stronger the larger the relative reduction in relatedness between current and future broods after the takeover.

There are two scenarios in which the interests of the group members are aligned. First, when every group member receives higher fitness when defeating the intruder (yellow area in figure 3). Second, when every group member receives higher fitness when not becoming involved in the contest (dark blue area in figure 3). There is one general scenario in which conflict arises within the group: when BS receives higher fitness returns from being involved in a contest that is won, but another group member receives lower returns (grey area in figure 3).

Switch points are generally found in the area of parameter space that corresponds to low chances of defeating the intruder (large *k*_1_ and/or large *s_I_*), low infanticide rates (small *μ*), long remaining brood-dependency periods (large *k*_3_), inefficient helpers (large *k*_2_) and low external survival rates (low *p*_surv_). Below, we discuss each parameter in terms of its effect on contest costs (cost of involvement) and on outcome costs (cost of takeover).

### (a) Low chances of defeating the intruder render contest costs high and takeover costs low

**Figure 4.**
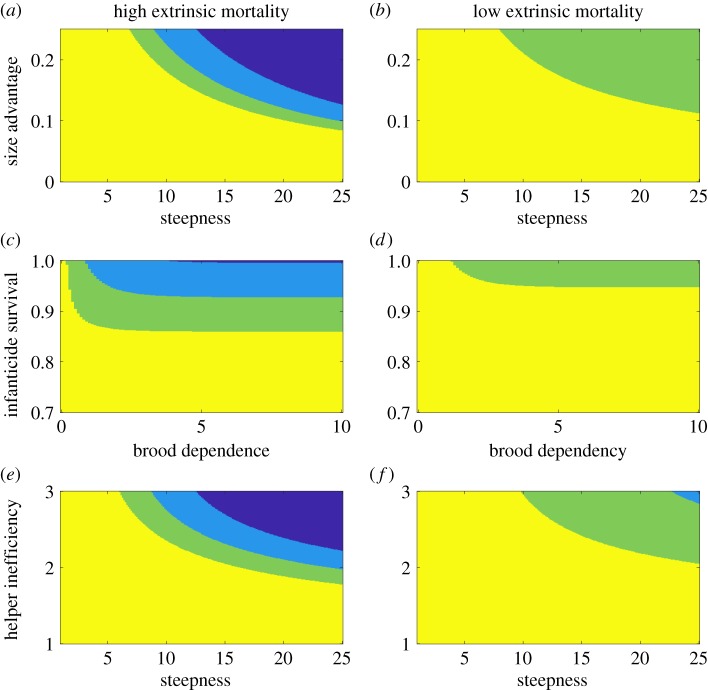
Effects of combinations of two parameters with external mortality (high and low) on the likelihood of within-group conflicts of interest. Area of parameter space where the interests of group members are aligned (yellow and dark blue area, denoted ‘no conflict’), where a conflict between BS and *H*_SO_ occurs (light blue), and where a conflict between BS and *H*_O_ occurs (green and light blue). Yellow area is where becoming involved in a contest and defeating the intruder is favourable for all group members; dark blue area is where not becoming involved in a contest and the intruder replaces BS is favourable for all group members. (*a,b*) Intruder's size advantage *s_I_* versus steepness of threshold curve *k*_1_; (*c,d*) infanticide survival 1 − *μ* versus duration of brood dependence *k*_3_; (*e,f*), helper inefficiency *k*_2_ versus steepness of threshold curve *k*_1_. Fixed parameters are: *s_I_* = 0.13, *μ* = 0.05, *k*_1_ = 20, *k*_2_ = 2, *k*_3_ = 10, (*a,c,e*) *p*_surv_ = 0.4 and (*b,d,f*) *p*_surv_ = 0.8. (Online version in colour.)

The parameters *s_I_* and *k*_1_ both affect the costs of involvement and outcome. On the one hand, the bigger the intruder, the more the group needs to get involved to win the contest, and consequently the lower resulting inclusive fitness for each group member (figure 2*a*; electronic supplementary material, C). Similarly, the required involvement for winning, and the accompanied costs, increase with *k*_1_ (which defines how fast the group involvement needed to overturn an intruder increases with the size advantage of the intruder, figure 2*b*). On the other hand, the bigger the intruder, the less it needs to invest in the contest, the higher its chances to survive to produce the second brood. All of which manifests in higher fitness returns for BO, *H*_O_ and *H*_SO_ in the case of a takeover (figure 2*a,b*; electronic supplementary material, C). Taken together, fitness returns from a takeover will exceed those from defeating the intruder at large values of *k*_1_ and *s_I_* for some group members but not yet for BS, which would lead to a within-group conflict. The area of parameter space without conflict is either at low *s_I_*- and *k*_1_-values, when the best option for all group members is to defeat the intruder (yellow area in figure 4*a,b*), or at large *s_I_*- and *k*_1_-values, when the best option for all group members is not to defend against the intruder (blue area in figure 4*a*). External mortality affects the positioning of these areas, and high mortality shifts the blue area (no defence by all) into lower regions of *s_I_* and *k*_1_.

### (b) Low infanticide rates keep takeover costs low and a long dependency period renders contest costs high

The parameters measuring infanticide and brood dependency both affect brood-related costs. A greater infanticide risk increases the cost of a takeover to group members because it lowers the fitness return derived from the current brood (figure 2*c*; electronic supplementary material, C). Thus, the higher the probability of infanticide, the more likely that BS and all other group members derive higher fitness returns from becoming involved in the contest. If infanticide risk is sufficiently low, such that BS also has higher fitness returns when the intruder takes over and does not kill the current brood, then the interests of group members can again be perfectly aligned, but this time in terms of not becoming involved in the contest. Contest costs, in terms of lower brood survival, increase with the duration of the dependency period, i.e. with *k*_3_ (figure 2*d*; electronic supplementary material, C). Thus, if *k*_3_ is sufficiently large, then a contest that needs a lot of involvement is less favourable for some group members, but not BS, and within-group conflict can be expected. If external mortality is high, no within-group conflict occurs at high infanticide rates (yellow area in figure 4*c*). At low infanticide rates, and when the current brood is somewhat dependent (i.e. *k*_3_ > 0), within-group conflict can occur (light blue and green area in figure 4*c*). If external mortality is low, the area of no conflict (yellow area in figure 4*d*) is larger.

### (c) Small and inefficient helpers increase contest costs

Small helpers are less efficient in defending than breeders and the group needs to raise more involvement to compensate this inefficiency. More involvement of the helper, however, decreases brood survival, and more involvement of the breeders lowers their survival and thus reproductive chances. Therefore, parameter *k*_2_, which measures a helper's inefficiency, increases the costs of defending against the intruder (figure 2*e*; electronic supplementary material, C). If *k*_2_ is sufficiently large, the fitness returns from not getting involved in the contest can exceed the returns from getting involved for some group members other than BS, leading to within-group conflict. As parameter *k*_2_ increases contest costs—in the same way as the parameters *s_I_* and *k*_1_—the interaction of *k*_2_ and either *s_I_* or *k*_1_ with external mortality rates is similar to that of *s_I_* and *k*_1_ (figure 4*e,f* shows the interaction between *k*_1_ and *k*_2_). That means the interests of group members are aligned at low *k*_1_- and *k*_2_-values, when the best option for all group members is to defeat the intruder, and at large *k*_1_- and *k*_2_-values, when the best option is to let the intruder take over.

### (d) External mortality lowers chances of future reproduction and increases the importance of fitness returns from the current brood

The higher the external mortality, the less likely it is that breeders (and the successful intruder) will survive to the next reproductive event. Expected fitness returns from the future brood thus decrease with external mortality (figure 2*f*; electronic supplementary material, C), and high mortality increases the area of parameter space in which within-group conflict can occur (figure 4). At the same time, external mortality increases the relative share of fitness returns from the current brood (see its interaction with brood-related parameters in figure 4*c,d*), which can render a costly involvement not worth the effort for group members other than BS. Thus, within-group conflict is more likely to occur when external mortality is high.

## 4. Discussion

This study presents a predictive model to assess when conflicts of interest arise among group members over contributions to defence against a single intruder seeking to seize a breeding position. In this scenario, the costs and benefits to individuals are likely to differ considerably on at least some occasions. Sufficiently high contest costs and sufficiently low takeover costs—or vice versa—can prevent within-group conflicts over defensive contributions. However, when contest and takeover costs are at intermediate levels, such that the cost–benefit ratios are below one for some group members (costs exceeding benefits) and above one (benefits exceeding costs) for others, we predict within-group conflict. Generally, an increased size advantage of the intruder, threshold for winning a contest, helper inefficiency and length of remaining brood dependency increase contest costs. Infanticide risk increases takeover costs, and external mortality amplifies the importance of the current brood to the overall lifetime fitness, thus acting as a catalyst for within-group conflict.

Our model, considering defence against a single intruder, results in some similar and some different predictions to those arising from the modelling of defence against a common threat to many group members. Collective-action problems (CAP) in the latter scenario mean that some variation in the level of defence is expected—for instance, dominant group members contributing more than subordinates to defensive actions—but similar input from dominants of both sexes is predicted [30]. In our model, this is mirrored by a breeder having the largest parameter space in which they favour defence against the outsider, but it is the challenged breeder which shows the greatest interest in defensive activity as they face the greatest potential threat; the interests of the two dominants are less strongly aligned than when defending a common good. As in defence against a common threat, where defensive contributions increase with increasing within-group relatedness [30], our model predicts that the helper's inclination to defend increases with its relatedness to the challenged breeder. Individual threats—rather than group threats—imply that in some circumstances, cooperative defence ceases to be beneficial for some but not all group members. Using our modelling approach, we identified when conflicts among group members arise over their contributions to defence against a single intruder. We predict a sequence of withdrawal of member's support for defending against the intruder; the sequence is determined by their relatedness to the challenged breeder (for the helpers) and the cost–benefit ratio (for the other breeder). Furthermore, we predict that high extrinsic mortality can increase within-group conflict—an aspect that has to our knowledge not been investigated within the CAP framework.

In our model, we focus on indirect benefits, but group members could also gain direct benefits. Some direct benefits are gained by all from being in a (larger) group, such as protection from predators or protection of resources; other direct benefits are more specific to helpers, such as having access to foraging resources, gaining experience of raising young, or inheriting a territory; and still others are more specific to breeders, such as keeping reproductive status or receiving parental care for their offspring [1,36]. Including direct benefits that apply to all group members and those that do not refer to inheriting or maintaining breeding status would probably have limited effect on the predictions of our model, because those benefits are often derived independently of contest outcome, especially given that group size does not change in our model. The case is somewhat different for a helper inheriting a breeding position. For example, the interests of a helper unrelated to the unchallenged breeder (*H*_S_) might no longer be fully aligned with the challenged breeder because the helper could be more likely to inherit the breeding position from the intruder, thus increasing the probability of within-group conflict. Direct benefits might also be increasingly important to consider with increasing group size; our model was based on two breeders and a single helper, a common scenario in facultative cooperatively breeding vertebrates [36], but could be extended to larger and more complex groups by adding an equation for each additional breeder and helper. In these additional equations, relatedness to the breeders would need to be adjusted, and the contribution of each group member to brood care and contest would need to be specified.

Another assumption of our current model is that future breeding success depends on the survival of the breeders but not that of the helper. The costs of a contest are thus higher if the involvement comes from the breeders, and involvement of the helper is therefore prioritized in our model. However, as a challenged breeder has the greatest risk from the intrusion (the potential loss of their breeding position), it is reasonable to assume that the challenged breeder gets most involved. Adding a further assumption on how helper survival affects future breeding success would not change the main conclusions. We expect that the additional assumption would predict somewhat higher breeder involvement such that other group members can contribute less, which would result in less within-group conflict. So, while the absolute position of switch points might shift, the relative order in which they arise would stay the same, and there would still be identifiable regions of parameter space where conflicts of interest between group members arise.

A few existing experimental studies have tested defensive contributions by group members faced with a single outsider seeking a breeding position and thus relate to the predictions of our model. For instance, Desjardins *et al*. [11] considered responses of *N. pulcher* cichlids to breeder-sized conspecific intruders (as well as predators) in captive-based experiments. In general, there was a stronger defence against a breeding threat by the relevant dominant group member, and greater defence by breeders than helpers; there was no evidence for a correlation between defence rates and the degree of size difference between defenders and intruders [11]. As another example, McComb *et al*. [59,60] have used playback experiments with wild African lions to simulate intrusions by outsiders, including both females and males. In the latter case, females show much stronger responses to unfamiliar cf. resident males, because the former represent an infanticidal risk to young cubs [60]. Such experimental studies are relatively rare and have tended to consider individual factors of importance in defensive decisions; our model suggests that the effect of different factors can interact in their influence on when and how much group members should contribute to defence against individual intruders.

To test hypotheses arising from our model, both between- and within-species empirical studies would be useful in the future. Phylogenetically controlled meta-analyses are increasingly used to test behavioural and evolutionary questions, not least because of the widespread availability of both relevant phylogenies and datasets containing information on ecological, life-history and social traits ([61] and references therein). Since our model pertains to various biological systems (see section Model), sufficient data from a range of species are probably available for such testing. As one potential example, infanticide occurs in only some species and even then is sex-specific, with males being the perpetrators in most cases [16]. As our model predicts that the probability of within-group conflict is largest when the infanticide risk is lowest, meta-analyses could consider comparisons of both species that do and do not exhibit infanticide as well as comparisons of infanticidal species where the intruder is or is not the infanticidal sex; group members of the species with higher infanticide risk should exhibit the same behaviour towards intruders, while those from lower infanticide risks should exhibit more varied responses to the intruder. In terms of within-species testing, ideally, there would be an experimental element potentially manipulating, for instance, the identity of the intruder or the relative size of the helper in a group. The cichlid fish *N. pulcher* provides an example of an excellent model for this kind of study, as they are group-living, face intrusions from individual outsiders, live and breed in captivity and have been shown to be amenable to out-group conflict manipulations [5,11].

In summary, our theoretical model suggests several different scenarios in which within-group conflict would be expected over defensive actions against individual conspecific intruders. Since within-group conflict is costly, conflict-management strategies are expected to evolve to minimize those costs. Indeed, there are now good examples of both aggressive and affiliative within-group behaviour before, during or after a conflict with outsiders [62]. Moving forward, there is a need to model these consequences of out-group conflict too. For now, our work can help to understand the variation seen in when and how much group members assist a breeder to repel an outsider. A full understanding of the evolution and maintenance of sociality requires greater integration between studies of within- and between-group interactions.

## Supplementary Material

Materials

## Data accessibility

Model details are provided in the electronic supplementary material. Code is provided on github: https://github.com/s-schindler/ConflictsWithinSocialGroups
