# Supplementary material for: Factors influencing within-group conflict over defence against conspecific outsiders seeking breeding positions
Source: Proc Biol Sci. 2018 Dec 19;285(1893):20181669. doi: 10.1098/rspb.2018.1669 (PMC6304047; doi:10.1098/rspb.2018.1669)
Supplement: Materials [file rspb20181669supp1.pdf]

Supplementary material for

Factors influencing within-group conflict over defence against conspecific outsiders seeking breeding positions.

Susanne Schindler & Andrew N. Radford

Contents

A Involvement in contest **2**
- A.1 Threshold of total group involvement needed to win the contest 2
- A.2 Involvement of group members 3
- A.3 Investment of intruder 3
- A.4 Consequences for brood 4

B Calculation of inclusive fitness **5**
- B.1 Costs to current brood 6
- B.2 Costs to future brood 7
- B.3 Total inclusive fitness 7

C When it is better to retreat than to fight? **9**
- C.1 Breeder BS 11
- C.2 Breeder BO 13
- C.3 Helper *H* 15

D Notation and parameters **17**

E Figures of modelling assumptions **19**

A Involvement in contest

A.1 Threshold of total group involvement needed to win the contest

To win a contest, we assume that a group's involvement has to exceed a certain threshold. We further assume that this threshold increases with the size advantage that the intruder has over the breeders. The parameter s_I captures the size advantage and ranges from 0 to 0.25, which is a realistic domain for natural systems. When an intruder is of the same size as both breeders ($s_I = 0$), we assume that the group has to raise a minimal amount of involvement $\epsilon = 0.1$ to defeat the intruder. To capture a broad range of systems for which different threshold slopes might exist, we introduce the parameter k_1. This parameter defines how fast the group's required minimal involvement increases with the size advantage of the intruder. The function describing the threshold is

$$\varphi_{\min}(s_I) = e^{(\ln \epsilon)\, e^{-k_1\, s_I}}, \tag{S1}$$

and figure S1 plots the threshold for various k_1-values. The larger k_1, the more group involvement is needed to defeat an intruder. In biological systems, an intruder's size advantage is probably below $s_I = 0.25$. For comparison, if $k_1 = 1$, 5, 10 or 20, the group involvement needed for defeating an intruder with $s_I = 0.25$ would be $\varphi_{\min} = 0.17$, 0.5, 0.8, or 0.98.

A.2 Involvement of group members

We assume that the group wins the contest when total group involvement, Φ — that is the sum of breeders' involvement and helper's involvement discounted by k_2 — is at least as large as the threshold of involvement needed to defeat an intruder:

$$\Phi = \varphi_{\text{BS}} + \varphi_{\text{BO}} + \frac{1}{k_2}\varphi_{\text{H}} \geq \varphi_{\text{min}}(s_I). \tag{S2}$$

We could assume that Φ has to exceed $\varphi_{\text{min}}(s_I)$ (i.e. '$>$'), but an established group usually has an advantage over an intruder because the contest happens on the group's territory (thus '$\geq$').

A.3 Investment of intruder

In our model, we focus on the case when the intruder is at least as large as the breeders ($s_I = 0$) or has a size advantage over the current breeders ($s_I > 0$), rather than when the intruder is smaller than the breeders. The investment of an intruder should be higher the smaller its relative size advantage s_I. In other words, when the intruder is very much larger than the breeders, a little bit of effort from the intruder suffices to be a challenge for the breeder. We therefore introduce a function which describes an intruder's investment φ_I and which decreases with s_I:

$$\varphi_I(s_I) = \frac{1}{1 + \frac{s_I}{\epsilon}}. \tag{S3}$$

We use $\epsilon = 0.1$ as above and with this function, we assume that an intruder's investment decreases steeply from the maximal investment possible $\varphi_I = 1$ (when $s_I = 0$, i.e. intruder is of the same size as the breeder) to a minimal investment of $\varphi_I = 0.29$ (when $s_I = 0.25$). Figure S2 shows the function $\varphi_I(s_I)$.

A.4 Consequences for brood

Involvement in contests defers energy resources away from the brood. Group members who are defending have less time to care for the current brood and might need time and resources for recovery in the aftermath of the contest. This matters less when the brood is nearly independent. To capture the impact of the remaining period of brood dependence and reduced care on the current brood, we introduce an exponentially decreasing function F with parameter k_3:

$$F(c, k_3) = c + (1 - c)\, e^{-k_3}. \tag{S4}$$

In this function, c is the maximal brood survival during a contest when the contest happens right at the beginning of the brood's dependent period. The parameter k_3 accounts for the duration of the remaining period in which the brood is dependent.

When parameter $k_3 = 0$ (i.e., the brood is independent and does not require any care), then $F(c) = 1$ (i.e., there are no expected costs to the current brood). If $k_3 \to \infty$ (i.e., more and more of the period of dependence remains and approaches the maximal period during which a brood of the

focal species requires care), then $F(c)$ approaches c. In other words, when the contest occurs at the end of the dependency period, then $k_3 = 0$, and when the contest occurs at the beginning of the dependency period, then $k_3 \geq 5$ or $k_3 \to \infty$. A plot of brood-survival during a contest, $F(c)$, as a function of k_3 is given in figure S3.

If the contest is lost, the intruder takes over and might kill the current brood. We capture the probability of infanticide with parameter μ. A value of $\mu = 0$ means that the intruder does not kill any member of the brood after successful takeover, whereas $\mu = 1$ means that the complete brood is killed upon takeover. Any value of μ between 0 and 1 means that the intruder kills the whole brood with probability μ and leaves the whole brood alive with probability $1 - \mu$. Mathematically, the result is equivalent to the scenario when a fraction μ of the surviving brood is killed by the successful intruder.

We capture the two types of costs to the brood – mortality through reduced care and through infanticide – by replacing c with $c\,(1-\mu)$ in function F. See figure S3 for how both costs depend on the duration of the remaining dependency period (i.e., on parameter k_3).

B Calculation of inclusive fitness

The expected fitness of group members is composed of two contributions: one contribution comes from seeing the current brood through to independence and one from producing another brood after the contest. We assume that brood size and brood survival is the same for each breeding pair, be it BO with BS or be it BO with intruder I. Thus, we do not need to include

parameters for brood size or offspring survival.

Brood-survival costs apply to current and future broods. In subsections B.1 and B.2, we estimate each of these costs, before adding them up in subsection B.3 to calculate the inclusive fitness values.

B.1 Costs to current brood

We compare two cases: 1) the contest is won by the group and the intruder defeated, and 2) the group does not get involved in the contest, the contest is lost by the group, and the intruder replaces BS. In case 1, to estimate the survival reduction of the brood resulting from the group's involvement in the contest, we average group involvement $\bar{\varphi} = (\varphi_{\mathrm{BS}} + \varphi_{\mathrm{BO}} + \varphi_{\mathrm{H}})/3$ and set survival of the current brood to $1 - \bar{\varphi}$. In doing so, we assume that all group members can provide parental care and each group member can equally perform the role of a carer. Entering this term as c into F (equation (S4)) and weighing it with relatedness coefficient r gives the expected fitness contribution arising from the current brood in the case of a won contest:

$$r\,F(1 - \bar{\varphi}, k_3). \tag{S5}$$

In case 2, the current brood loses the care which would have been provided by BS. Two of the three original group members are still available to provide care. As BS will be lost, we can set φ_{BS} to 1, the maximal value, and calculate $1 - \bar{\varphi} = \frac{3-(1+\varphi_{\mathrm{BO}}+\varphi_{\mathrm{H}})}{3} = \frac{2-\varphi_{\mathrm{BO}}-\varphi_{\mathrm{H}}}{3}$. The intruder is not expected to contribute to care for the brood as it is not related to it. Some species show infanticide, so we have to weight $\frac{2-\varphi_{\mathrm{BO}}-\varphi_{\mathrm{H}}}{3}$ with the probability to survive

infanticide: $\frac{2-\varphi_{\mathrm{BO}}-\varphi_{\mathrm{H}}}{3}(1-\mu)$. Entering this term as c into F and weighing it with relatedness coefficient r gives the expected fitness contribution coming from the current brood in the case of a takeover:

$$r\,F\left(\frac{2-\varphi_{\mathrm{BO}}-\varphi_{\mathrm{H}}}{3}(1-\mu), k_3\right). \tag{S6}$$

B.2 Costs to future brood

Regardless of whether the contest is won or lost, the breeding pair (either BS and BO or BO and I) produce a new brood. The probability that a breeder or the intruder survive to the reproductive event is diminished by their involvement in the contest. That means, in the case of winning the contest, the probability that BS and BO reproduce is $(1-\varphi_{\mathrm{BS}})\,(1-\varphi_{\mathrm{BO}})\,p_{\mathrm{surv}}^2$. And in the case of losing the contest, the probability of BO reproducing with the intruder is $(1-\varphi_{\mathrm{BO}})\,(1-\varphi_I(s_I))\,p_{\mathrm{surv}}^2$. Thus, the fitness contribution arising from the future brood and weighted with the relatedness coefficient, is

$$r\,(1-\varphi_{\mathrm{BS}})\,(1-\varphi_{\mathrm{BO}})\,p_{\mathrm{surv}}^2 \qquad \text{in case of defeating I,} \tag{S7}$$

$$r\,\big(1-\varphi_{\mathrm{BO}}\big)\,\big(1-\varphi_I(s_I)\big)\,p_{\mathrm{surv}}^2 \qquad \text{in case of a takeover.} \tag{S8}$$

B.3 Total inclusive fitness

In this section we combine the fitness contributions arising from the current and the new brood. This gives the expected inclusive fitness in the two cases: 1 (group wins) with required involvement levels $\varphi_{\mathrm{BS}}^{\mathrm{won}}$, $\varphi_{\mathrm{BO}}^{\mathrm{won}}$, and $\varphi_{\mathrm{H}}^{\mathrm{won}}$, and case 2 (group loses).

Let $w^{\text{won}}(\varphi_{\text{BS}}, \varphi_{\text{BO}}, \varphi_{\text{H}})$ and $w^{\text{lost}}(\varphi_{\text{BS}}, \varphi_{\text{BO}}, \varphi_{\text{H}})$ denote respective inclusive fitness values with the specified levels of involvement for case 1 (group wins, i.e., defeats I) and case 2 (group loses, i.e. I replaces BS). For BS, we calculate its inclusive fitness in case 1 by summing equation (S5) and (S7) and inserting the relatedness coefficient $r = 1/2$. In case 2, inclusive fitness of BS consists only of the contribution from the current brood, i.e. from equation (S6):

$$w_{BS}^{\text{won}}(\varphi_{\text{BS}}^{\text{won}}, \varphi_{\text{BO}}^{\text{won}}, \varphi_{\text{H}}^{\text{won}}) = \frac{1}{2}\big(F(1-\bar{\varphi}^{\text{won}}, k_3) + (1-\varphi_{\text{BS}}^{\text{won}})\,(1-\varphi_{\text{BO}}^{\text{won}})\,p_{\text{surv}}^2\big) \tag{S9}$$

$$w_{BS}^{\text{lost}}(\varphi_{\text{BS}}, \varphi_{\text{BO}}, \varphi_{\text{H}}) = w_{BS}^{\text{lost}}(\varphi_{\text{BO}}, \varphi_{\text{H}}) = \frac{1}{2}F\left(\frac{2-\varphi_{\text{BO}}-\varphi_{\text{H}}}{3}(1-\mu), k_3\right). \tag{S10}$$

For BO and the helper, we similarly sum the contributions from the current and new brood and substitute the appropriate coefficient of relatedness. While for BO, r_{BO} is 1/2 in both cases, the relatedness coefficient of the helper to the current brood, r_H, and to the brood produced with the intruder, r'_H, depend on its relationship to BS and BO (values in table S1):

$$w_{BO}^{\text{won}} = \frac{1}{2}\big(F(1-\bar{\varphi}^{\text{won}}, k_3) + (1-\varphi_{\text{BS}}^{\text{won}})\,(1-\varphi_{\text{BO}}^{\text{won}})\,p_{\text{surv}}^2\big) \tag{S11}$$

$$w_{BO}^{\text{lost}} = \frac{1}{2}\Big(F\big(\tfrac{2-\varphi_{\text{BO}}-\varphi_{\text{H}}}{3}(1-\mu), k_3\big) + (1-\varphi_{\text{BO}})\,\big(1-\varphi_I(s_I)\big)\,p_{\text{surv}}^2\Big) \tag{S12}$$

$$w_{H}^{\text{won}} = r_H\,\big(F(1-\bar{\varphi}^{\text{won}}, k_3) + (1-\varphi_{\text{BS}}^{\text{won}})\,(1-\varphi_{\text{BO}}^{\text{won}})\,p_{\text{surv}}^2\big) \tag{S13}$$

$$w_{H}^{\text{lost}} = r_H\,F\big(\tfrac{2-\varphi_{\text{BO}}-\varphi_{\text{H}}}{3}(1-\mu), k_3\big) + r'_H\,(1-\varphi_{\text{BO}})\,(1-\varphi_I)\,p_{\text{surv}}^2. \tag{S14}$$

C When it is better to retreat than to fight?

The optimal action (getting involved, at what level, or not at all) secures the highest inclusive fitness value, so we have to check for each group member whether its inclusive fitness is higher when defeating the intruder or when letting it take over BS's position. Group winning (defeating the intruder) is preferable if and only if $w^{\text{won}}(\varphi_{\text{BS}}^{\text{won}}, \varphi_{\text{BO}}^{\text{won}}, \varphi_{\text{H}}^{\text{won}}) > w^{\text{lost}}(\varphi_{\text{BS}}, \varphi_{\text{BO}}, \varphi_{\text{H}})$ for any φ_{BS}, φ_{BO}, and φ_{H}. If both terms are equal, we assume that members of the group would prefer to keep the current situation so we can replace '$>$' with '$\geq$':

$$w^{\text{won}}(\varphi_{\text{BS}}^{\text{won}}, \varphi_{\text{BO}}^{\text{won}}, \varphi_{\text{H}}^{\text{won}}) \geq w^{\text{lost}}(\varphi_{\text{BS}}, \varphi_{\text{BO}}, \varphi_{\text{H}}). \tag{S15}$$

When inclusive fitness values from defeating the intruder fall below the fitness values from letting the intruder take over, retreating is evolutionarily better than fighting. That means, a group member will defend against the intruder as long as inequality (S15) is met. The condition in equation (S15) can be met for some group members, but not for others.

As involvement is costly, inclusive fitness decreases with the involvement of any group member, i.e. $w^{\text{lost}}(\varphi_{\text{BS}}, \varphi_{\text{BO}}, \varphi_{\text{H}}) \leq w^{\text{lost}}(0,0,0)$. In other words, the highest fitness values in the case of losing the contest occur when the group does not defend at all. Therefore, the condition for when winning is preferable over losing amounts to:

$$w^{\text{won}}(\varphi_{\text{BS}}^{\text{won}}, \varphi_{\text{BO}}^{\text{won}}, \varphi_{\text{H}}^{\text{won}}) \geq w^{\text{lost}}(0,0,0). \tag{S16}$$

A challenged breeder will usually get involved in repelling an intruder when the intruder obtains the breeder's reproductive monopoly. So, realistically φ_{BS} is not zero but positive ($\varphi_{\mathrm{BS}} > 0$). However, $w^{\mathrm{lost}}(\varphi_{\mathrm{BO}}, \varphi_{\mathrm{H}})$ does not depend on φ_{BS} as BS is replaced and its missing contribution to brood care is already accounted for.

The fitness return expected from the current brood decreases equally with the involvement of any group member (case 1: BS, BO, H; case 2: BO, H), but the returns from the future brood, and thus also from overall inclusive fitness (equations S9–S14), are most sensitive to the involvement of the breeders. This is because the breeder's involvement lowers the fitness return from the future brood, whereas the helper's involvement does not. It is thus in the interest of every group member that the helper gets involved as much as is needed to defeat the intruder. Only if the helper's involvement is not sufficient to win the contest, breeders should also become involved. This is clearly not what we see in nature, where often – though not always – breeders defend against intruders more than helpers. However, as we have discussed above, any involvement of group members, be it breeders or the helper, will lower the right-hand side of inequality (S16), thus making it easier to met the inequality and thus less likely for a conflict of interest within the group to occur. Focusing on fitness returns from losing *without* group involvement thus gives the broadest parameter range possible in which a conflict between group members can occur.

Mathematically, the optimal involvement of each group member in the case where all aim to win, i.e. defeat the intruder (denoted with superscript

'won') is then:

$$\varphi_{\mathrm{H}}^{\mathrm{won}} = \min(1, k_2\, \varphi_{\min}(s_I)) \tag{S17}$$

$$\varphi_{\mathrm{BS}}^{\mathrm{won}} = \varphi_{\mathrm{BO}}^{\mathrm{won}} = \frac{\varphi_{\min}(s_I) - \frac{1}{k_2}\varphi_{\mathrm{H}}^{\mathrm{won}}}{2}. \tag{S18}$$

Please note, because $\varphi_{\min}(s_I)$ is smaller than or equal to 1, but Φ can exceed 1, the group will definitely win the contest if group members get involved at the levels stated in equations (S17–S18). In the next sections, we will use equations (S9–S14, S17, S18) and discuss when inequality (S16) is met for each group member.

C.1 Breeder BS

Inserting (S9–S10) into (S16) and canceling the factor 1/2 which appears on both sides, gives the condition when BS prefers to keep its breeding position, i.e. to win the contest:

$$F(1 - \bar{\varphi}^{\mathrm{won}}, k_3) + (1 - \varphi_{\mathrm{BS}}^{\mathrm{won}})\,(1 - \varphi_{\mathrm{BO}}^{\mathrm{won}})\, p_{\mathrm{surv}}^2 \;\geq\; F\left(\frac{2}{3}(1-\mu), k_3\right). \tag{S19}$$

Surprisingly, BS might not always want to win the contest and we will formally derive this result from (S19). We will use a property of function F, namely that F increases with its first argument. That is, if we can show that if $1 - \bar{\varphi}^{\mathrm{won}}$ is bigger than $\frac{2}{3}(1-\mu)$, then $F\big(1 - \bar{\varphi}^{\mathrm{won}}, k_3\big) > F\big(\frac{2}{3}(1-\mu), k_3\big)$, and then inequality (S19) is fulfilled.

Let us first focus on the case $\varphi_{\mathrm{H}}^{\mathrm{won}} = k_2\, \varphi_{\min}(s_I) < 1$. In this case $\varphi_{\mathrm{BS}}^{\mathrm{won}} = \varphi_{\mathrm{BO}}^{\mathrm{won}} = 0$, and thus $1 - \bar{\varphi}^{\mathrm{won}} = 1 - \frac{\varphi_{\mathrm{H}}^{\mathrm{won}}}{3}$. Let us look at the relationship

between this term and $\frac{2}{3}(1-\mu)$:

$$1-\frac{\varphi_{\text{H}}^{\text{won}}}{3} > \frac{2}{3}(1-\mu) \tag{S20}$$

$$\Leftrightarrow \quad 3-\varphi_{\text{H}}^{\text{won}} > 2-2\,\mu \tag{S21}$$

$$\Leftrightarrow \quad 1-\varphi_{\text{H}}^{\text{won}} > -2\,\mu. \tag{S22}$$

The last equation (S22) is fulfilled, because $\varphi_{\text{H}}^{\text{won}} < 1$ as we assumed, and thus (S20) is fulfilled, and consequently (S19) is fulfilled. So if $\varphi_{\text{H}} = k_2\,\varphi_{\min}(s_I) < 1$, that is, the helper can single-handedly defeat the intruder alone, then BS prefers to win the contest.

Let us now focus on the case $\varphi_{\text{H}}^{\text{won}} = 1$ and $\varphi_{\text{BS}} = \varphi_{\text{BO}} = \frac{\varphi_{\min}(s_I)-\frac{1}{k_2}}{2}$, that is the breeders might become involved in the contest. Inequality (S16) becomes:

$$F\left(\frac{2-\varphi_{\min}+\frac{1}{k_2}}{3}, k_3\right) + \left(1-\frac{\varphi_{\min}(s_I)-\frac{1}{k_2}}{2}\right)^2 p_{\text{surv}}^2 \geq F\left(\frac{2}{3}(1-\mu), k_3\right), \tag{S23}$$

which is equivalent to

$$F\left(\frac{2-\varphi_{\min}+\frac{1}{k_2}}{3}, k_3\right) - F\left(\frac{2}{3}(1-\mu), k_3\right) + \frac{1}{4}\left(2-\varphi_{\min}(s_I)+\frac{1}{k_2}\right)^2 p_{\text{surv}}^2 \geq 0. \tag{S24}$$

We will use a property of the F-function:

$$F(a,k_3)-F(b,k_3) = (a-b)\,(1-e^{-k_3}), \tag{S25}$$

and (S24) becomes

$$\frac{2\,\mu - \varphi_{\min}(s_I) + \frac{1}{k_2}}{3}\,(1 - e^{-k_3}) + \frac{1}{4}\left(2 - \varphi_{\min}(s_I) + \frac{1}{k_2}\right)^2 p_{\text{surv}}^2 \geq 0. \quad \text{(S26)}$$

The second term of (S26) is non-negative, so let us focus on the first term. If $2\,\mu - \varphi_{\min}(s_I) + \frac{1}{k_2} > 0$, then (S26) is fulfilled and BS gains higher fitness returns from defeating the intruder. Put in words, if infanticide probability is high, the intruder is small and easy to defeat (s_I or $\varphi_{\min}(s_I)$ small), or the helper is efficient (k_2 close to 1), then BS prefers to win the contest with the intruder.

If the first term of (S26) is negative, then a small value of k_3 (current brood is nearly independent) can ensure (S26) is met. However, if infanticide probability is low, the intruder hard to defeat (either large s_I or high $\varphi_{\min}(s_I)$), the helper inefficient (large k_2), the remaining dependency period long (large k_3), and survival uncertain (low p_{surv}), then (S26) might not be met. Under the listed conditions BS can receive higher fitness returns by sidestepping the conflict and letting the intruder take over, rather than harming the current brood with lower parental care due to the group getting involved in the contest. For example, in our model, BS profits from resigning from the breeding position without fighting when $k_1 = 20$, $k_2 = 2$, $k_3 = 10$, $s_I = 0.23$, $\mu = 0.02$, and $p_{\text{surv}} = 0.5$.

C.2 Breeder BO

We obtain the condition when BO receives higher fitness returns from defeating the intruder than from letting the intruder take over from inserting

(S11–S12) into (S16) and cancelling factor 1/2:

$$F\big(1-\bar{\varphi}^{\mathrm{won}}, k_3\big) + (1-\varphi_{\mathrm{BS}}^{\mathrm{won}})\,(1-\varphi_{\mathrm{BO}}^{\mathrm{won}})\,p_{\mathrm{surv}}^2 \geq F\big(\tfrac{2}{3}(1-\mu), k_3\big) + \big(1-\varphi_I(s_I)\big)\,p_{\mathrm{surv}}^2. \tag{S27}$$

We distinguish the two cases $\varphi_{\mathrm{H}}^{\mathrm{won}} = k_2\,\varphi_{\min}(s_I) < 1$ and $\varphi_{\mathrm{H}}^{\mathrm{won}} = 1$ as we did above for BS. In the first case, we find again that $F\big(1-\bar{\varphi}^{\mathrm{won}}, k_3\big) > F\big(\frac{2}{3}(1-\mu), k_3\big)$ and that $(1-\varphi_{\mathrm{BS}}^{\mathrm{won}})\,(1-\varphi_{\mathrm{BO}}^{\mathrm{won}})\,p_{\mathrm{surv}}^2 = p_{\mathrm{surv}}^2 \geq \big(1-\varphi_I(s_I)\big)\,p_{\mathrm{surv}}^2$. Thus, (S27) is fulfilled. In other words, if the helper can single-handedly defeat the intruder, then BO, just as BS, prefers to win the contest with the intruder.

In the second case, $\varphi_{\mathrm{H}}^{\mathrm{won}} = 1$ and $\varphi_{\mathrm{BS}} = \varphi_{\mathrm{BO}} = \frac{\varphi_{\min}(s_I)-\frac{1}{k_2}}{2}$. Inequality (S27) then reads:

$$F\left(\frac{2-\varphi_{\min}+\frac{1}{k_2}}{3}, k_3\right) - F\left(\frac{2}{3}(1-\mu), k_3\right) + \ldots$$
$$+\left(\frac{1}{4}\left(2-\varphi_{\min}(s_I)+\frac{1}{k_2}\right)^2 - \big(1-\varphi_I(s_I)\big)\right) p_{\mathrm{surv}}^2 \geq 0. \tag{S28}$$

We use the property (S25) of F, and (S28) simplifies to

$$\frac{2\,\mu-\varphi_{\min}(s_I)+\frac{1}{k_2}}{3}\,(1-e^{-k_3}) + \left(\frac{1}{4}\left(2-\varphi_{\min}(s_I)+\frac{1}{k_2}\right)^2 - \big(1-\varphi_I(s_I)\big)\right) p_{\mathrm{surv}}^2 \geq 0. \tag{S29}$$

If $2\,\mu-\varphi_{\min}(s_I)+\frac{1}{k_2} < 0$ then it no longer suffices when the brood is independent ($k_3 = 0$) to make (S29) hold, because a large intruder (meaning low

$\varphi_I(s_I)$) can make the second term of (S29) negative. Conditions that make (S29) hold are: high infanticide probability, small intruder (low $\varphi_{\min}(s_I)$ and large $\varphi_I(s_I)$), efficient helpers (small k_2), and a long dependency period (large k_3). The opposite conditions can cause BO to receive higher fitness returns when the intruder replaces BS. Specifically, when there is a small infanticide probability, large intruder (large $\varphi_{\min}(s_I)$ and low $\varphi_I(s_I)$), small helper (large k_2), and a short dependency period (small k_3).

Although this set of conditions is qualitatively the same as those that make BS prefer a takeover, condition (S29) is quantitatively harder to meet than (S26). This means the probability for BO to prefer a takeover is higher than for BS and there is a section of parameter space where BS prefers to defeat the intruder while BO does not. For example, when $k_1 = 20$, $k_2 = 2$, $k_3 = 10$, $\mu = 0.02$, and $p_{\text{surv}} = 0.5$, BS receives higher fitness returns from a takeover when the intruder is 1.23 times larger than the breeders, while BO would receive higher fitness returns when the intruder is only 1.1 times larger.

C.3 Helper H

We obtain the condition when helpers receive higher fitness returns from defeating the intruder than from letting the intruder take over from inserting (S13–S14) into (S16) and entering the appropriate relatedness factors from table S1:

Helper H_S:

$$\frac{1}{4}\left(F\big(1-\bar{\varphi}^{\text{won}}, k_3\big) + \left(1-\varphi_{\text{BS}}^{\text{won}}\right)\left(1-\varphi_{\text{BO}}^{\text{won}}\right)p_{\text{surv}}^2\right) \geq \frac{1}{4}\,F\big(\tfrac{2}{3}(1-\mu), k_3\big) \qquad \text{(S30)}$$

Helper H_O:

$$\frac{1}{4}\left(F(1-\bar{\varphi}^{\text{won}}, k_3)+(1-\varphi_{\text{BS}}^{\text{won}})\,(1-\varphi_{\text{BO}}^{\text{won}})\,p_{\text{surv}}^2\right) \geq \frac{1}{4}\,F(\tfrac{2}{3}(1-\mu), k_3)+\frac{1}{4}\,\Big(1-\varphi_I(s_I)\Big)\,p_{\text{surv}}^2 \tag{S31}$$

Helper H_{SO}:

$$\frac{1}{2}\left(F(1-\bar{\varphi}^{\text{won}}, k_3)+(1-\varphi_{\text{BS}}^{\text{won}})\,(1-\varphi_{\text{BO}}^{\text{won}})\,p_{\text{surv}}^2\right) \geq \frac{1}{2}\,F(\tfrac{2}{3}(1-\mu), k_3)+\frac{1}{4}\,(1-\varphi_I(s_I))\,p_{\text{surv}}^2 \tag{S32}$$

We cancel the factor $\frac{1}{4}$ from both sides of inequalities (S30–S31) and find that (S30) is the same as (S19), and (S31) is the same as (S27). Therefore, the conditions of H_S's switch point are the same as for BS, and the conditions for H_O the same as for BO.

For helper H_{SO}, we multiply (S32) with 2, use property (S25) and obtain:

$$\left(1-\bar{\varphi}^{\text{won}}-\frac{2}{3}(1-\mu)\right)\,(1-e^{-k3})+\left((1-\varphi_{\text{BS}}^{\text{won}})\,(1-\varphi_{\text{BO}}^{\text{won}})-\frac{1}{2}\,(1-\varphi_I(s_I))\right)\,p_{\text{surv}}^2 \geq 0 \tag{S33}$$

We distinguish the two cases $\varphi_{\text{H}}^{\text{won}} = k_2\,\varphi_{\text{min}}(s_I) < 1$ and $\varphi_{\text{H}}^{\text{won}} = 1$ as we did above for BS and BO. In the first case, we find again that (S20) holds and that (S33) is fulfilled. In the second case, $\varphi_{\text{H}}^{\text{won}} = 1$ and $\varphi_{\text{BS}} = \varphi_{\text{BO}} = \frac{\varphi_{\text{min}}(s_I)-\frac{1}{k_2}}{2}$. Inequality (S33) then reads:

$$\frac{2\,\mu-\varphi_{\text{min}}(s_I)+\frac{1}{k_2}}{3}\,(1-e^{-k_3})+\left(\frac{1}{4}\left(2-\varphi_{\text{min}}(s_I)+\frac{1}{k_2}\right)^2-\frac{1}{2}\,\big(1-\varphi_I(s_I)\big)\right)\,p_{\text{surv}}^2 \geq 0.$$

(S34)

Condition (S34) is very similar to (S29), the only difference is factor $\frac{1}{2}$ in front of $1 - \varphi_I(s_I)$, which makes (S34) easier to meet than (S29). However, the conditions under which H_{SO} receives higher fitness returns when the intruder takes over are qualitatively the same conditions as for BO, but that the conditions are quantitatively relaxed. This means, that H_{SO} withdraws its support for BS later than B_O and H_O, but earlier than H_S.

D Notation and parameters

Parameter	Description	Function	Domain	Values used for figures
ϵ	Minimal involvement of group needed to defeat an intruder of the same size as the breeders		$\epsilon > 0$	0.1
μ	Probability that successful intruder will kill current brood upon takeover		$\mu \in [0,1]$	$\mu \in [0,1]$
k_1	Steepness of φ_{min}		$k_1 > 0$	(0,25]
k_2	Inefficiency of helper		$k_2 \geq 1$	[1,3]
k_3	Duration of remaining period in which brood is dependent		$k_3 \geq 0$	[0,10]
p_{surv}	Adult survival rate		$p_{\text{surv}} \in [0.1, 1]$	
s_I	Size advantage of intruder over breeders		$s_I \geq 0$	$s_I \in [0, 0.25]$
$\varphi_I(s_I)$	Investment of intruder with size advantage s_I	$\frac{1}{1+\frac{s_I}{\epsilon}}$	$\varphi_I(s_I) \in (0,1]$	
φ_{H}	Involvement of helper	$\varphi_{\text{H}} = \min(1, k_2\, \varphi_{\text{min}})$	$\varphi_{\text{H}} \in [0,1]$	
φ_{BS}	Involvement of breeder (same sex as intruder)	$(\varphi_{\text{min}} - \varphi_{\text{H}})/2$	$\varphi_{\text{BS}} \in [0,1]$	
φ_{BO}	Involvement of breeder (other sex than intruder)	$(\varphi_{\text{min}} - \varphi_{\text{H}})/2$	$\varphi_{\text{BO}} \in [0,1]$	
Φ	Total group involvement	$\varphi_{\text{BS}} + \varphi_{\text{BO}} + \frac{1}{k_2}\varphi_{\text{H}}$	$\Phi \in [0,3]$	
$\varphi_{\text{min}}(s_I)$	Minimal group involvement needed to defeat an intruder with s_I	$e^{(\ln \epsilon)\, e^{-k_1 s_I}}$	$\varphi_{\text{min}} \in [\epsilon, 1]$	
$F(c, k_3)$	Survival rate of current brood	$c + (1-c)\, e^{-k_3}$	$F \in (c, 1]$	
c	Minimal brood survival upon takeover if intrusion occurs at the start of the dependency period.		$c \in [0,1]$	0.5
$r_{BS}\,(r'_{BS})$	Relatedness coefficient between BS and current brood before a takeover (and to future brood after a takeover)		$r_{BS}, r'_{BS} \in [0,1]$	0.5 (0)
$r_{BO}\,(r'_{BO})$	Relatedness coefficient between BO and current brood before a takeover (and to future brood after a takeover)		$r_{BO}, r'_{BO} \in [0,1]$	0.5 (0.5)
$r_H\,(r'_H)$	Relatedness coefficient between helper and current brood before a takeover (and to future brood after a takeover)		$r_H, r'_H \in [0,1]$	see below
$r_{SO}\,(r'_{SO})$	Relatedness of a helper who is an offspring of both breeders to current brood (to future brood after takeover)			0.5 (0.25)
$r_O\,(r'_O)$	Relatedness of a helper who is an offspring or sibling of BO (but not of BS) to current brood (to future brood after takeover)			0.25 (0.25)
$r_S\,(r'_S)$	Relatedness of a helper who is an offspring or sibling of BS (but not of BO) to current brood (to future brood after takeover)			0.25 (0)
$r_{\text{unrel}}\,(r'_{\text{unrel}})$	Relatedness of a helper who is unrelated to either of BS or BO to current brood (to future brood after takeover)			0 (0)

Table S1

Notation, parameters, functions, and values used in this study.

E Figures of modelling assumptions

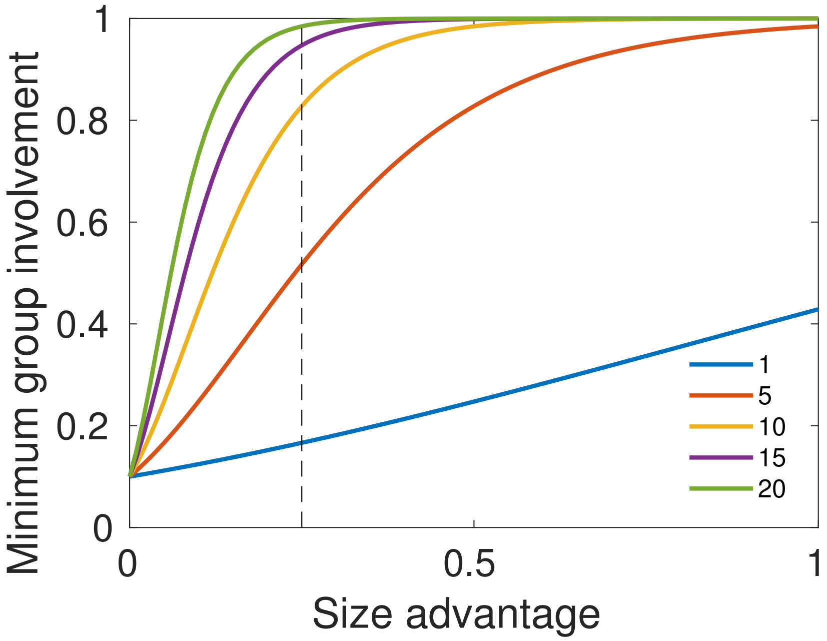

Figure S1: Involvement needed, φ_{min}, to defeat an intruder with size advantage s_I. The dashed line indicates when $s_I = 0.25$, which is the maximal size advantage that an intruder can have in realistic biological scenarios. The values of parameter k_1 are given in the legend.

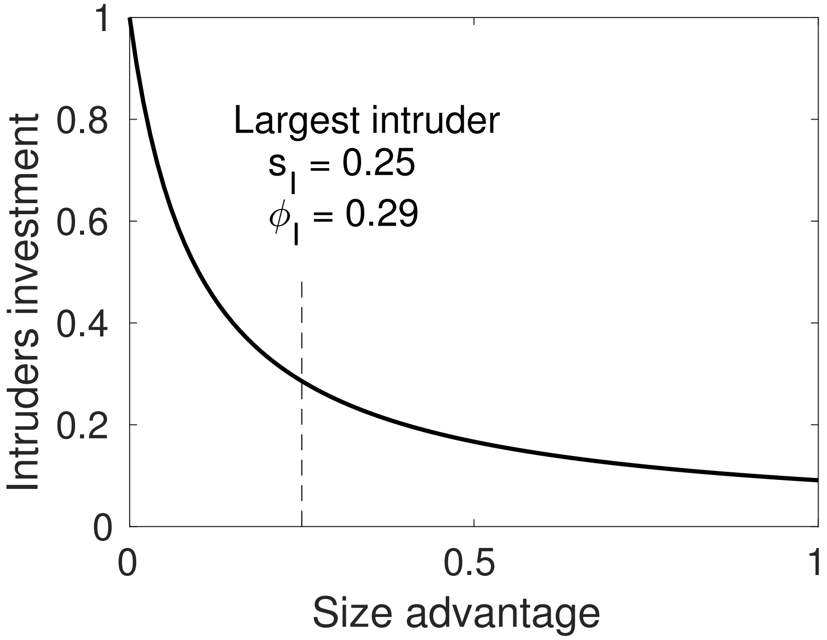

Figure S2: Investment in contest by an intruder with size advantage s_I. The dashed line indicates when $s_I = 0.25$, which is the maximal size advantage that an intruder can have in realistic biological scenarios.

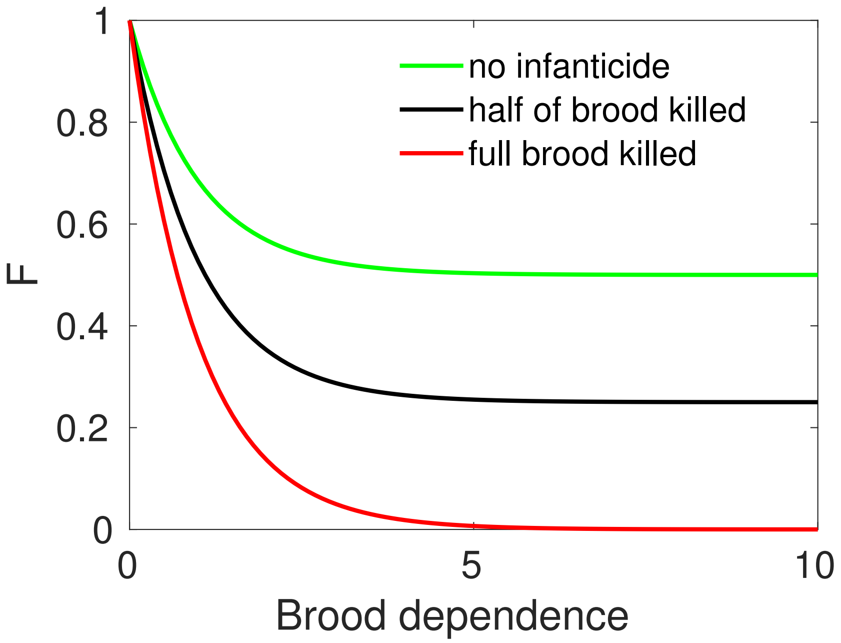

Figure S3: The costs to the current brood that occur through the involvement of a group in contest decrease exponentially with k_3. A low value of k_3 means that the current brood is nearly independent, because the contest occurred towards the end of the parental care period. A larger value ($k_3 \geq 5$) means that the contest occurred at the beginning of the parental care period; the brood is still dependent for a while. The plot shows survival of the current brood when there is no infanticide ($\mu = 0$, green), an infanticide probability of 50% ($\mu = 0.5$, black), and when infanticide by the new breeder is certain ($\mu = 1$, red). The other parameter used in this plot is $c = 0.5$.
